# Demenz mit Lewy-Körpern: alte und neue Erkenntnisse – Teil 1: Klinik und Diagnostik

**DOI:** 10.1007/s00115-023-01576-3

**Published:** 2023-12-13

**Authors:** Richard Dodel, Daniela Berg, Thomas Duning, Elke Kalbe, Philipp T. Meyer, Alfredo Ramirez, Alexander Storch, Dag Aarsland, Frank Jessen

**Affiliations:** 1 Lehrstuhl für Geriatrie, Universität Duisburg-Essen, Virchowstraße 171, 45147 Essen, Deutschland; 2 Neurologische Klinik, Universität Kiel, Kiel, Deutschland; 3 Neurologische Klinik, Universität Münster, Münster, Deutschland; 4 Medizinische Psychologie, Neuropsychologie und Gender Studies & Centrum für Neuropsychologische Diagnostik und Intervention (CeNDI), Universität Köln, Köln, Deutschland; 5 Klinik für Nuklearmedizin, Universitätsklinikum Freiburg, Medizinische Fakultät, Albert-Ludwigs-Universität Freiburg, Freiburg, Deutschland; 6 Klinik und Poliklinik für Psychiatrie und Psychotherapie, Universität Köln, Köln, Deutschland; 7 Klinik für Neurologie, Universität Rostock, Rostock, Deutschland; 8 Centre for Age-Related Medicine (SESAM), Stavanger University Hospital, Stavanger, Norwegen; 9 Institute of Psychiatry, Psychology, and Neuroscience, King’s College London, London, Großbritannien

**Keywords:** Neuropsychologie, Genetik, Epidemiologie, Zerebrale Bildverarbeitung, Parkinson-Krankheit mit Demenz, Neuropsychologie, Genetics, Epidemiology, Cerebral imaging, Parkinson’s disease with dementia

## Abstract

**Hintergrund:**

Die Demenz mit Lewy-Körpern (DLK) ist nach der Alzheimer-Krankheit die zweithäufigste neurodegenerative Demenzerkrankung. Patienten mit DLK haben oft eine schlechte Prognose, mit schlechteren Ergebnissen als Patienten mit der Alzheimer-Krankheit in Bezug auf wichtige Parameter wie Lebensqualität, Belastung der Pflegepersonen, gesundheitsbezogene Kosten, Einweisungshäufigkeit in Krankenhäuser und Pflegeheime, kürzerer Zeitraum bis zur schweren Demenz und eine geringere Überlebensrate. Die DLK wird häufig fehldiagnostiziert und ist oft unterbehandelt. Daher ist es von entscheidender Bedeutung, die DLK so früh wie möglich zu diagnostizieren, um eine optimale Versorgung und Behandlung zu gewährleisten.

**Ziel der Arbeit:**

In diesem Übersichtsartikel sollen die wichtigsten neuen Erkenntnisse zu diagnostischen Instrumenten, der Epidemiologie und Genetik der Demenz mit Lewy-Körpern zusammengetragen werden.

**Ergebnisse:**

Es existieren präzise klinischen Diagnosekriterien für die DLK, die eine ätiologische Zuordnung zulassen. Bildgebende Verfahren kommen standardmäßig bei der DLK zum Einsatz, insbesondere auch, um nicht neurodegenerative Ursachen auszuschließen. Insbesondere nuklearmedizinische Verfahren besitzen eine hohe diagnostische Aussagekraft.

**Diskussion:**

Die Diagnose beruht in erster Linie auf der klinischen Symptomatik, obwohl die Entwicklung von In-vivo-Neurobildgebung und Biomarkern den Umfang der klinischen Diagnose sowie die Erforschung dieser verheerenden Krankheit verändert.

## Abstract (en)

**Background:**

Dementia with Lewy bodies (DLB) is the second most common neurodegenerative dementia after Alzheimer’s disease. Patients with DLB often have a poor prognosis, with worse outcomes than patients with Alzheimer’s disease in terms of important parameters, such as quality of life, caregiver burden, health-related costs, frequency of hospital and nursing home admissions, shorter time to severe dementia, and lower survival. The DLB is frequently misdiagnosed and often undertreated. Therefore, it is critical to diagnose DLB as early as possible to ensure optimal care and treatment.

**Objective:**

The aim of this review article is to summarize the main recent findings on diagnostic tools, epidemiology and genetics of DLB.

**Results:**

Precise clinical diagnostic criteria exist for DLB that enable an etiologic assignment. Imaging techniques are used as standard in DLB, especially also to exclude non-neurodegenerative causes. In particular, procedures in nuclear medicine have a high diagnostic value.

**Discussion:**

The diagnosis is primarily based on clinical symptoms, although the development of in vivo neuroimaging and biomarkers is changing the scope of clinical diagnosis as well as research into this devastating disease.

Die Diagnose einer Demenz mit Lewy-Körpern (DLK) ist präzise definiert und richtet sich nach den (wissenschaftlichen) Konsensuskriterien, die 2017 neu aufgelegt wurden und eine Unterscheidung in 6 Kategorien treffen [1]: Haupt‑, Kern-, unterstützende klinische Merkmale, hinweisende Biomarker/unterstützende Biomarker sowie Befunde, die eine Diagnose wenig wahrscheinlich machen, und die zeitliche Abfolge der Symptome. Weiter unterscheidet man die diagnostische Sicherheit zwischen möglicher und wahrscheinlicher DLK [2]. Die Genauigkeit der neuen Diagnosekriterien sind bisher nur unzureichend evaluiert; für die vorangegangenen Kriterien konnte eine hohe Spezifität (93 %), aber niedrige Sensitivität (73 %) aufgezeigt werden [3].

Die Diagnose der DLK und insbesondere die Abgrenzung von der Alzheimer-Krankheit, kann im Frühstadium schwierig sein, wenn Kernmerkmale der Erkrankung nicht oder nur ungenügend ausgeprägt sind. Etwa die Hälfte der Patienten mit DLK-Pathologie zeigt klinisch das typische Bild einer DLK. Folglich gibt es eine hohe Anzahl an Fällen, die eine atypische Präsentation mit einer koexistierenden Alzheimer-Pathologie zeigen und somit klinisch schwer einzuordnen sind (das typische klinische Bild korreliert „direkt mit dem Ausmaß der Lewy-Körper-Pathologie und invers mit dem Ausmaß der gleichzeitigen AD-Pathologie“). Andererseits weisen auch viele Alzheimer-Patienten ein gewisses Maß an DLK-Pathologie auf, meist in den Amygdalae [4]. Weiter konnte eine retrospektive Anwendung der Konsensuskriterien auf eine Autopsie-verifizierte Gruppe zeigen, dass Fälle in der Braak-Alzheimer-Klassifikation im Stadium 5 und 6 der Tangle-Pathologie wahrscheinlich nicht korrekt klinisch diagnostiziert werden [5]. Daher ist es nicht verwunderlich, dass Personen mit DLK fälschlicherweise als Alzheimer-Kranke diagnostiziert werden, was negative Folgen haben kann, obwohl die diagnostische Sensibilität durch einfache Mittel zusätzlich zu Biomarkern wie der Dopamintransporter-SPECT erhöht werden kann [6].

Neben den genannten „McKeith-Kriterien“ wurde erstmalig in dem DSM-5-Manual die leichte und schwere neurokognitive Störung aufgrund einer DLK aufgenommen. Zur Sensitivität und Spezifität dieser Kriterien sind bisher noch keine Daten veröffentlicht worden.

Ganz neu wird die Demenz in den neuen Parkinson-Kriterien der Movement Disorder Society konzeptualisiert [7]. Die DLK sollte entsprechend nicht länger als Ausschlusskriterium betrachtet werden, sondern die Demenz wird als ein Symptom im natürlichen Verlauf der Parkinson-Krankheit (PK) angesehen. Entsprechend wird die DLK als Teil des Spektrums der Parkinson-Krankheit im Sinne eines Subtyps konzeptualisiert.

Die zeitliche Abfolge motorischer und kognitiver Symptome spielt im Konzept der Lewy-Körper-Erkrankung eine wichtige Rolle: Die kognitiven Störungen treten vor oder nahezu gleichzeitig mit der Entwicklung der motorischen Parkinson-Symptomatik auf, zumindest aber nicht später als ein Jahr nach Erstmanifestation des motorischen Parkinson-Syndroms („Einjahresregel“; [1]). Treten kognitive Symptome danach, im Rahmen einer seit Jahren bestehenden Parkinson-Krankheit auf, spricht man von einer Parkinson-Krankheit mit Demenz.

## Prodromale Stadien

Von der Arbeitsgruppe der internationalen Movement Disorders Society wird sehr klar die Auffassung formuliert, dass es sich bei der DLK um eine Erkrankung im Spektrum der Parkinson-Syndrome handelt, wobei in der Regel neben den für die Parkinson-Erkrankung typischen α‑Synuclein-Ablagerungen häufig noch vaskuläre und/oder Aβ-Pathologien vorliegen. Kürzlich hat eine weitere Arbeitsgruppe um McKeith Kriterien für das prodromale Stadium vorgelegt [8].

Auch wenn die Einordnung der DLK noch immer kontrovers diskutiert wird [9], überwiegen die Parallelen von PK und DLK in neuropsychologischen Befunden, Art der Demenz, Präsentation der motorischen und nichtmotorischen Symptome, Bildgebung (z. B. SPECT und PET), Genetik und Pathologie. Da die DLK somit Teil des Parkinson-Spektrums ist, teilt sie auch die bei der Parkinson-Erkrankung mittlerweile in vielen Studien belegte prodromale Phase. Dies wird besonders evident durch die Tatsache, dass eine REM-Schlaf-Verhaltensstörung (RBD) ein typisches prodromales Symptom aller Synucleinopathien (PK, DLK, Multi-System Atrophie [MSA]) ist. In einer großen, multizentrischen Studie [10] konnte gezeigt werden, dass knapp 75 % der von RBD-Betroffenen nach 12 Jahren klinische Symptome einer Synucleinopathie ausgebildet hatten. In 56,5 % dominierte ein Parkinson-Syndrom, in 43,5 % eine Demenz als erstes klinisches Symptom der neurodegenerativen Erkrankung. Auch in anderen Studien konnte gezeigt werden, dass die Kombination von RBD und neuropsychologischen Auffälligkeiten die klinische Manifestation einer DLK wahrscheinlich machen [11]. Der Goldstandard für die Diagnose einer RBD ist die Polysomnographie (PSG). Allerdings weisen ca. 25 % der DLK-Patient:innen keine RBD-typischen Befunde in der PSG auf, sodass auch innerhalb der DLK von Subtypen auszugehen ist.

## Neuropsychologische Testung

Laut Konsensuskriterien muss für eine klinische Diagnose einer DLK – den üblichen Demenzkriterien entsprechend – ein progressiver Abbau kognitiver Funktionen gegeben sein, der die Aktivitäten des täglichen Lebens z. B. im sozialen oder beruflichen Kontext beeinträchtigt [1]. Während weiterhin Beeinträchtigungen in der Aufmerksamkeit, exekutiven Funktionen und visuoperzeptiven Fähigkeiten als prominent angegeben werden, gehören Gedächtnisstörungen nicht unbedingt zu den frühen Symptomen, können aber später hinzukommen.

Wie auch bei anderen Demenzdiagnostikempfehlungen üblich, kann die neuropsychologische Diagnostik zur Abklärung einer DLK in zwei Stufen erfolgen: der sog. „Level-I“-Diagnostik, die der zeitökonomischen Feststellung und gegebenenfalls Schweregradeinteilung kognitiver Beeinträchtigungen dient, und einer „Level-II“-Diagnostik mit einer elaborierten neuropsychologischen Testbatterie, die auch die Bildung eines kognitiven Profils erlaubt.

In einer umfassenden Übersichtsarbeit von Walker et al. werden für die Level-I-Diagnostik verschiedene Screeningverfahren empfohlen, wobei nur der MoCA als generisches Screening und der PANDA als krankheitsspezifisches (eigentlich für Parkinson-Patienten entwickeltes) Instrument in deutschsprachigen Versionen verfügbar sind. McKeith et al. empfehlen weiterhin den Mini Mental Status Test (MMST), der weniger sensitiv als z. B. der MoCA ist, sich allerdings zur Verlaufsbeobachtung („Tracking“) bis in spätere Krankheitsstadien gut zu eignen scheint [1]. Für den MoCA konnte gezeigt werden, dass dieser sogar sensitiv genug ist, um im Prodromalstadium einer DLK kognitive Veränderungen anzuzeigen.

Für die Level-II-Diagnostik werden viele verschiedene Tests zur Erfassung der unterschiedlichen kognitiven Domänen empfohlen, wobei die einschlägige Literatur Inhomogenitäten aufweist, indem teilweise unterschiedliche Tests genannt werden und die Domänen anders geclustert sind als z. B. in den Empfehlungen zur Diagnostik der PK-MCI bzw. PKD (Parkinson-Krankheit mit Demenz) und damit auch die Zuordnung einzelner Tests zu einzelnen Domänen unklar ist. Aufgrund des spezifischen kognitiven Profils der DLK erscheint die Erfassung von visuokognitiven sowie Aufmerksamkeitsfähigkeiten, die besonders betroffen sind, in der Demenzdiagnostik oft aber gegenüber der Testung von Gedächtnis- und exekutiven Funktionen zurücksteht, besonders wichtig.

Zur Erfassung kognitiver Fluktuationen, die insbesondere zur Abgrenzung zu anderen Demenzen wesentlich ist, stehen verschiedene klinische Skalen zur Verfügung mit Fragen, die entweder über Angehörige oder vom Kliniker beantwortet werden [12]. Der Angehörigenfragebogen „Dementia Cognitive Fluctuation Scale“, für den 17 Items aus vormals bestehenden Skalen zusammengestellt wurden, eignet sich für die zeitökonomische und zuverlässige Einschätzung wohl sehr gut [13]. Ein Problem stellt allerdings die bislang mangelnde Verfügbarkeit im deutschsprachigen Raum dar.

Eine zuverlässige Differenzialdiagnose mittels des neuropsychologischen Profils etwa zwischen der DLK und der PKD ist aufgrund der hohen Ähnlichkeit kaum möglich, klinisch aber auch weniger relevant, da sich die Diagnosen über das Zeitkriterium mit der „Einjahresregel“ zum relativen Beginn kognitiver bzw. motorischer Symptomen definiert. Dagegen besteht in der Unterscheidung der Alzheimer Demenz und DLK eine sog. „doppelte Dissoziation“, indem bei der Alzheimer-Krankheit typischerweise das Gedächtnis stärker als visuoperzeptive oder visuokonstruktive Leistungen betroffen ist und bei der DLK das umgekehrte Profil zu beobachten ist; für den Einzelfall insbesondere bei weiter fortgeschrittenen Demenzen ist dieses Profil jedoch wenig scharf. Mit dem „Free and Cued Selective Reminding Test“ lassen sich Alzheimer-typische Gedächtnisstörungen von denen bei anderen Demenzen – auch der DLK – besonders gut abbilden. Bei der DLK sind besonders schlechte visuell-räumliche Leistungen prädiktiv für einen stärkeren kognitiven Abbau bei DLK-Patienten.

Für eine zukünftig verbesserte neuropsychologische Diagnostik der DLK wäre eine Homogenisierung der Testempfehlungen – und auch der Domänenzuordnungen – mit Spezifikation der im deutschsprachigen Raum verfügbaren Instrumente wichtig. Eine Übersetzung und Validierung der im Rahmen des „UK National Institute for Health Research DIAMONDS“ entwickelten standardisierten Toolkits zur Verbesserung der DLK-Diagnose [14], die auch die neuropsychologischen Beeinträchtigungen systematisch abfragt, wäre hierfür äußerst sinnvoll.

## Epidemiologie

Lange Zeit wurde die DLK als zweithäufigste Demenz bezeichnet [15] und in der älteren neuropathologischen Literatur wird eine Häufigkeit pathologischer Veränderungen im Sinne von Lewy-Körpern bei etwa 15–20 % aller Demenzfälle zitiert. Allerdings stehen nur vereinzelt populationsbasierte neuropathologische Studien [16] zu dieser Fragestellung zur Verfügung [17], die eine differenzierte Bewertung der Häufigkeit der verschiedenen demenziellen Erkrankungen bedingt durch die erheblichen Überschneidungen bei den Pathologien zulassen (in der Honolulu Asia Aging Study beispielsweise konnten 33 % der dementen Personen keinem der vier primären pathogenen Prozesse oder einer Kombination davon zugeordnet werden – vaskuläre Läsionen, AD-Läsionsmuster, Hippokampussklerose und kortikale Lewy-Körper; [18]).

Neuere klinisch-epidemiologische Daten zeigen aber, dass die Häufigkeit etwa 5 % aller Demenzfälle beträgt [19].

Die Inzidenzraten reichen von 0,5–1,6 pro 1000 Personenjahre. In den Inzidenzstudien fanden sich etwa 3,2–7,1 % aller Demenzfälle. Punkt- und Periodenprävalenzschätzungen reichten von 0,02–63,5 pro 1000 Personen. Zunehmende Prävalenzschätzungen wurden mit zunehmendem Alter berichtet. In den Prävalenzstudien entfielen von allen Demenzfällen 0,3–24,4 % auf die DLK. Das Verhältnis Männer zu Frauen beträgt bei der DLK 1,5:1. Das mittlere Alter bei Diagnosestellung liegt in einer amerikanischen Studie bei ca. 70,8 ± 9,4 Jahren bzw. in einer taiwanesischen Studie bei 76,3 ± 9,8 Jahren [20, 21]. Die Überlebenszeit betrug in beiden Studien ca. 7 Jahre.

## Genetik

Das Verständnis der Genetik bei der DLK ist derzeit noch unzureichend. Die DLK hat eine geschätzte genetische Komponente (Heritabilität) von ca. 36 %, was darauf hindeutet, dass genetische Varianten einen Einfluss auf pathophysiologische Pathways haben, die bei der DLK eine Rolle spielen. Drei Gene sind als Risikofaktor für die DLK etabliert oder an der Pathologie der DLK beteiligt [22, 23].

### Apolipoprotein-E-Gen (***APOE***).

Das APOE-ɛ4-Allel ist ein bekannter Risikofaktor für die AD. Auch für die DLK konnte eine genetische Korrelation gezeigt werden. DLK-Patienten, die Genträger des ɛ4-Allels sind, haben eine kürzere Prognose quoad vitam im Vergleich zu Personen, die dieses Allel nicht besitzen.

### α-Synuclein-Gen (***SNCA***).

Sechs verschiedene Missense-Mutationen sowie Multiplikationen (Du‑/Triplikationen des SCNA-Gens) sind bekannt, die eine Parkinson-Krankheit (PK) verursachen können [24]. Genomische Triplikationen sind häufiger mit der DLK und PKD assoziiert. Pathogene Mutationen in SNCA sind sehr selten und können zu einem breiten phänotypischen Spektrum, das von der PK über DLK, atypische Parkinson-Syndrome und bis hin zu den frontotemporalen Lobärdegenerationen reicht, führen.

### Glukozerebrosidase-Gen (***GBA***).

Homozygote Mutationen im GBA-Gen verursachen die Gaucher-Krankheit; aber auch heterozygote Träger dieser Varianten zeigten eine höhere Prävalenz für das Auftreten von Parkinson-Symptomen. Ebenfalls für DLK-Patienten konnte eine erhöhte Odds Ratio mit einem Wert von 8,28 (95 %-KI: 4,78–14,88) nachgewiesen werden, wobei GBA-Varianten mit einem früheren Krankheitsbeginn und früheren Tod verknüpft sein können [25].

Die Einführung von genomweiten Assoziationsstudien (GWAS) und Next-Generation-Sequencing-Ansätzen hat die Identifizierung von Suszeptibilitätsfaktoren bei komplexen Erkrankungen wie der DLK beschleunigt. Im Falle der ersten GWAS bei DLK könnte der Beitrag von *SNCA, APOE* und *GBA* als bestimmende Risikofaktoren für DLK bestätigt werden [26, 27]. GWAS in DLK replizierten für *SNCA* beispielsweise, dass PK und DLK differenzielle Assoziationsprofile im *SNCA*-Lokus haben [28]. Bezüglich der Risikomodulation konnte für einige Varianten in *GBA* ein eindeutiger Zusammenhang festgestellt werden [29]. Zur weiteren Darstellung der verschiedenen Kandidatenrisikogene verweisen wir auf kürzlich erschiene Übersichtsartikel [23].

Ferner führten erste Sequencing-Ansätze bei seltenen familiären DLK-Fällen oder nichtverwandten sporadischen DLK-Fällen zu der Identifizierung seltener kodierender Varianten in weiteren Genen, darunter Presenilin 1 (*PSEN1*), Presenilin 2 (*PSEN2*), Amyloidvorläuferprotein (*APP*), β‑Synuclein (*SNCB*), „leucine-rich repeat kinase 2“ (*LRRK2*), „microtubule-associated protein tau“ (*MAPT*; [30]). Während die ersten drei Gene mit familiären Fällen von AD in Verbindung gebracht werden, tragen die anderen drei Gene seltene kodierende Varianten, die eine familiäre Form von PK verursachen oder mit dem Risiko für das Auftreten einer PK in Verbindung gebracht wurden.

Die Entdeckung seltener Varianten in AD-Genen (*PSEN1, PSEN2* und *APP*) in Fällen von Demenz könnte jedoch zum Teil auch auf die falsche Diagnose zurückzuführen sein, insbesondere wenn ein neuropathologischer Befund nicht möglich war. Das gleichzeitige Auftreten von LK-Pathologie bei Alzheimer ist häufig und kann den Krankheitsphänotyp in Richtung DLK beeinflussen [31]. Darüber hinaus sind einige der in diesen Studien identifizierten Varianten von unbekannter Pathogenität, was weitere Fragen zur Beteiligung dieser Gene an der DLK aufwirft. Andererseits haben genetische Studien die Hypothese gestützt, dass Suszeptibilitätsgene, die bei Alzheimer und Parkinson eine Rolle spielen, auch zum DLK-Risiko beitragen könnten. So berichteten Cia et al. (2021) über die bisher größte GWAS-Studie zur DLK, die 2591 Patienten und 4027 Kontrollpersonen umfasste [28], und nutzten dabei genomweite Sequenzierungsdaten. In dieser Studie wurden fünf genetische Signale identifiziert, die ein genomweites Signifikanzniveau erreichten, wodurch der Beitrag von *APOE, SNCA* und *GBA* in der Kausalkaskade der DLK bestätigt wurde und zwei neue Signale, eines in *BIN1* und das andere auf *TMEM175*, gemeldet wurden. Während *BIN1* mit der pathogenen Kaskade in Verbindung gebracht wird, die die Amyloid-β- und Tau-Pathologie bei Alzheimer moduliert, kodiert *TMEM175* ein in den Lysosomen lokalisiertes Protein, und das Gen befindet sich an einem bekannten PK-Risikolokus (PMID: 33589841). Darüber hinaus zeigten verwertete genetische Daten in Form eines genetischen Risikoscores, dass DLK Risikoprofile und -wege mit AD und PK teilt [28].

Trotz dieser interessanten Ergebnisse ist die Stichprobengröße, die in den aktuellen GWAS für DLK verwendet wird, immer noch begrenzt, um häufige Varianten mit kleinen Effektgrößen zu detektieren, sodass die meisten der häufigen Suszeptibilitätsvarianten für DLK unbekannt bleiben. Demzufolge sind jetzt größere Studien erforderlich, die Histopathologie, Genetik und klinische Befunde kombinieren, um zu bestätigen, ob Gene, die familiäre Formen der Alzheimer-Krankheit verursachen, auch für die DLK verantwortlich sind oder nicht.

## Bildgebung

### Strukturelle Bildgebung

Computertomographie (CT) und Magnetresonanztomographie (MRT) kommen standardmäßig bei der DLK und der Diagnostik anderer Demenzformen zum Einsatz, insbesondere auch, um nicht neurodegenerative Ursachen auszuschließen. Die Atrophie der grauen Substanz zeigt sich bei der AD vorwiegend in den medialen Strukturen des Temporallappens und den temporoparietalen Assoziationskortizes; diese Strukturen sind bei der DLK weit weniger stark betroffen als bei der AD und können zur Abgrenzung verwendet werden. Weitere Veränderungen, wie z. B. in den subkortikalen Strukturen, Unterschiede in der Atrophierate oder der kortikalen Dicke in bestimmten Regionen eignen sich noch nicht für die differenzialdiagnostische Abgrenzung im klinischen Alltag. Ähnliches gilt für den Verlust des „Schwalbenschwanzzeichens“ („swallow tail sign“) in MRT-Sequenzen sensitiv für Speichereisen (SWI) der Substantia nigra [32].

Insgesamt legen diverse Vergleichsstudien aber eine höhere diagnostische Aussagekraft nuklearmedizinischer Verfahren nahe [33], sodass auf diese im Folgenden näher eingegangen werden soll.

### Nuklearmedizinische Bildgebung

**Figure 1:**
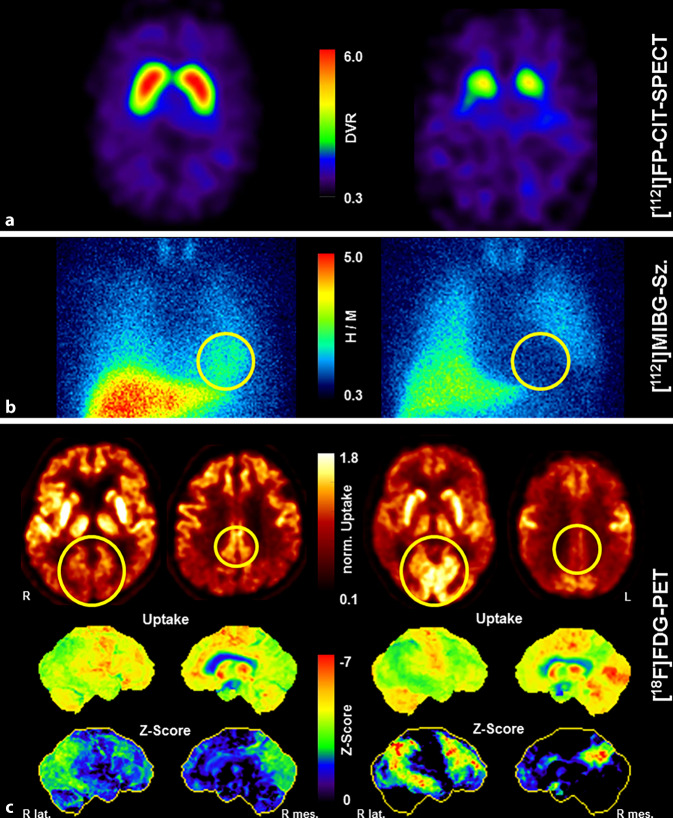

Die SPECT mit dem Dopamintransporter(DAT)-Liganden [^123^I]FP-CIT dient der Visualisierung und Quantifizierung der Integrität des nigrostriatalen dopaminergen Systems. Sie spiegelt die präsynaptische striatale DAT-Dichte und damit das Ausmaß einer möglichen Degeneration der Substantia nigra, Pars compacta wider (s. Übersicht Abb. 1). Die DAT-Dichte ist bei DLK häufig vermindert, während sie bei Patienten mit Demenz infolge AD in der Regel normal ist, sodass diese Untersuchung insbesondere in der Differenzialdiagnose dieser beiden Erkrankungen hilfreich ist (Abb. 1a). Übereinstimmend mit einer Metaanalyse, die eine hohe Sensitivität (87 %) und Spezifität (94 %) der [^123^I]FP-CIT-SPECT gegenüber der klinischen Referenzdiagnose beschrieb (DLK vs. Non-DLK) [34], bestätigen auch mehrere Arbeiten mit Post-mortem-Verifikation eine sehr hohe diagnostische Genauigkeit (Sensitivität: 80–92 %; Spezifität 83–100 %; [35–37]). Die DAT-SPECT wird daher gemäß den aktuellen klinischen Kriterien für DLK als „indikativer Biomarker“ eingestuft [1]. Dies bedeutet z. B., dass bereits 1 Kernsymptom der DLK bei verminderter DAT-Verfügbarkeit die klinische Diagnose einer „wahrscheinlichen DLK“ bzw. eine verminderter DAT-Verfügbarkeit alleine die Diagnose einer „möglichen DLK“ rechtfertigt. Es ist darauf hinzuweisen, dass eine unauffällige DAT-SPECT eine DLK aber nicht ausschließt (anders als bei der PK laut den aktuellen PK-Kriterien [7]), da die Substantia nigra bei einer vorrangig kortikalen Lewy-Körper-Pathologie in Einzelfällen ausgespart sein kann [38]. Bei fortbestehendem klinischem Verdacht auf eine DLK ist daher eine SPECT-Verlaufskontrolle zu erwägen, um eine oft im Verlauf von wenigen Jahren noch auftretende nigrostriatale Degeneration zu sichern [39]. Jüngste Arbeiten unterstreichen auch die hohe prognostische Bedeutung der [^123^I]FP-CIT-SPECT (medianes Überleben 4,4 vs. 8,1 Jahre bei demenzieller Erkrankung mit/ohne nigrostriataler Degeneration) [40].

Das radioaktiv markierte Noradrenalinanalog [^123^I]MIBG wird zur szintigraphischen Darstellung der postganglionären sympathischen Innervation des Herzens eingesetzt, welche bei DLK und PK im Gegensatz zur AD und den sog. atypischen Parkinson-Syndromen (APS) oft bereits im frühen Krankheitsstadium reduziert ist (Abb. 1b). In einer frühen Metaanalyse wurde eine sehr hohe Sensitivität und Spezifität dieser Methode (98 % bzw. 94 %) zur Abgrenzung der DLK gegenüber anderen demenziellen Erkrankungen beschrieben [42]. Es ist jedoch darauf hinzuweisen, dass hier die klinische Diagnose nach damaligen DLK-Kriterien als Referenz diente, die selbst nur eine eingeschränkte diagnostische Genauigkeit besitzt (ca. 80 % [43]). In einer jüngeren, größeren japanischen Multicenterstudie mit 3‑jährigem Follow-up wurde die Sensitivität und Spezifität der [^123^I]MIBG-Szintigraphie mit 77 % bzw. 97 % für die Differenzierung zwischen wahrscheinlicher DLK und AD angegeben (Fälle mit möglicher DLK wurden ausgeschlossen) [44]. Bisher gibt es nur wenige Daten zur notwendigen Validierung der [^123^I]MIBG-Szintigraphie gegenüber der Post-mortem-Neuropathologie, die jedoch eine sehr hohe Sensitivität (> 90 %) der [^123^I]MIBG-Szintigraphie nahelegen, auch wenn einzelne DLK-Patienten eine normale sympathische Innervation des Herzens zeigen können [45]. Wie die DAT-SPECT wird die [^123^I]MIBG-Szintigraphie daher in den neuen DLK-Kriterien auch als „indikativer Biomarker“ aufgeführt [1].

Die PET mit dem Glukoseanalogon [^18^F]Fluordesoxyglukose ([^18^F]FDG) dient der quantitativen Darstellung des zerebralen Glukosestoffwechsels, welcher unmittelbar an die regionale neuronale Funktion gekoppelt ist. Die [^18^F]FDG-PET besitzt als Biomarker der Neurodegeneration (sog. „[N]“ Biomarker) einen etablierten Stellenwert in der Demenzdiagnostik [77]. Bei der DLK zeigt sich typischerweise wie bei der AD ein temporoparietaler sowie ein darüber hinausgehender und diagnostisch wegweisender okzipitaler Hypometabolismus. Im Gegensatz zur AD sind bei der DLK ferner der Precuneus und posteriore Gyrus cinguli relativ ausgespart (sog. „cingulate island sign“; Abb. 1c). Laut einer systematischen Literaturübersicht beträgt die Sensitivität und Spezifität der [^18^F]FDG-PET zur Diagnostik der DLK vs. AD ca. 70–92 % bzw. 74–100 %, was auch eine Studie mit Post-mortem-Verifikation einschließt (Sensitivität 90 %, Spezifität 80 %; [46, 47]). Zudem besitzt die [^18^F]FDG-PET auch eine hohe prognostische Bedeutung [48]. In den aktualisierten DLK-Kriterien wird die [^18^F]FDG-PET daher als „supportiver Biomarker“ aufgeführt. Erwähnt wird als solcher auch die Perfusions-SPECT, doch weist diese eine gegenüber der zeitgemäßen PET eine deutlich reduzierte Bildqualität auf und wurde bereits in früheren Arbeiten als der [^18^F]FDG-PET unterlegen beschrieben [49]. Nach Meinung der Autoren sollte die Perfusions-SPECT nur in Ausnahmefällen angewandt werden.

Im Vergleich erscheinen sowohl die [^123^I]FP-CIT-SPECT als auch die [^123^I]MIBG-Szintigraphie der [^18^F]FDG-PET für die Differenzierung DLK vs. AD überlegen (Metaanalyse: [50]; DAT-SPECT vs. [^18^F]FDG-PET: [33, 51]). In Head-to-head-Vergleichen weisen die beiden erstgenannten Methoden eine vergleichbare diagnostische Eignung [52, 53] bzw. die [^123^I]FP-CIT-SPECT einen geringen Vorteil gegenüber der [^123^I]MIBG-Szintigraphie auf [54, 55]. Für die Anwendung der [^123^I]MIBG-Szintigraphie ist ferner nachteilig, dass zahlreiche Einflussfaktoren auf das Untersuchungsergebnis zu beachten sind (z. B. diverse Medikamente und in dieser Altersgruppe häufige Erkrankungen wie Diabetes, Polyneuropathie, diverse Herzerkrankungen, etc.), während die [^123^I]FP-CIT-SPECT ein sehr robustes und bereits rein visuell ausreichend genau beurteilbares Verfahren ist [56].

Kombinationen der beiden Methoden scheinen den einzelnen Verfahren überlegen zu sein [52, 54, 55], doch sind Mehrfachuntersuchungen im Hinblick auf die Kosten und Belastungen der Patienten nur bedingt umsetzbar. Eine Stratifizierung nach klinischen Variablen erscheint hier möglicherweise sinnvoller, so bietet sich eine [^123^I]FP-CIT-SPECT vor allem beim Vorliegen eines Parkinson-Syndroms an, während die [^123^I]MIBG-Szintigraphie häufiger beim Vorliegen von RBD einen pathologischen Befund ergibt [55].

Kommen bei im Vordergrund stehender kognitiver Einschränkung bzw. Parkinson-Syndrom weitere Differenzialdiagnosen in Betracht (z. B. FTD bzw. ein APS), kann es durchaus sinnvoll sein, im ersten Schritt eine relativ günstigere [^18^F]FDG-PET durchzuführen [57, 58]. Einerseits liegt bei diesen Differenzialdiagnose ebenfalls häufig (FTD) bzw. regelhaft (APS) eine pathologische [^123^I]FP-CIT-SPECT vor [56]. Andererseits erlauben unauffällige Befunde in der [^123^I]FP-CIT-SPECT bzw. [^123^I]MIBG-Szintigraphie keine weiterführende Differenzierung, wie die [^18^F]FDG-PET es bei dieser Konstellation erlaubt (z. B. zwischen AD, FTD und fehlender Neurodegeneration) [57, 58]. Wird bei klinisch weiterhin unklaren Fällen nach der [^18^F]FDG-PET noch eine [^123^I]FP-CIT-SPECT ergänzt, erlaubt diese in der Regel eine sehr sichere Differenzialdiagnostik (siehe z. B. nahezu perfekte Gruppentrennung von DLK und AD [33]).

Neben diesen klinisch etablierten Verfahren möchten wir auf zwei interessante aktuelle Entwicklungen hinweisen: Während die Entwicklung von Bildgebungsbiomarkern von α‑Synuclein-Ablagerungen bisher noch nicht erfolgreich ist, stehen neben dem prototypischen Liganden [^11^C]PIB bereits seit einigen Jahren umfassend validierte, auch kommerziell verfügbare PET-Liganden zur Verfügung (Florbetapir, Florbetaben, Flutemetamol), die eine sehr genaue In-vivo-Detektion von β‑Amyloid(Aβ)-Plaques erlauben (sog. „A“-Biomarker). Zudem schreitet die Entwicklung von Liganden fibrillärer Tau-Ablagerungen (v. a. der gemischten 3‑ und 4‑Repeat-Isoformen wie bei AD) rasch voran; sog. „T“-Biomarker. Die Bildgebung mit diesen Liganden erscheint aus verschiedenen Gründen überaus vielversprechend.

Passend zu neuropathologischen Befunden, dass die DLK häufig zusammen mit einer Alzheimer-Kopathologie auftritt, weisen rund 60 % der DLK-Patienten (aber nur rund 35 % der PK-Patienten mit Demenz) einen positiven Befund in der Amyloid-PET auf [59]. Jüngere Studien legen nahe, dass eine begleitende Amyloidpathologie die Ausprägung und das Voranschreiten der kognitiven Einschränkung bei DLK verstärkt [60, 61] In einer kürzlich erschienenen PET-/Neuropathologiestudie korrelierte die [^11^C]PIB-Bindung ferner nicht nur eng mit der Ausprägung von Aβ-Plaques, sondern auch mit dem Braak-Stadium der Tau-Neurofibrillen-Bündel und dem Lewy-Körper-Score [62]. Zusammengenommen ist dies ggf. auf eine synergistische Wirkung von α‑Synuclein, Aβ und auch Tau zurückzuführen [61, 62]. Die hohe Frequenz amyloidpositiver Befunde in der üblichen binären visuellen PET-Befundung bei DLK (bzw. DLK/AD) schränkt deren diagnostische Eignung zur Trennung DLK vs. AD ein, auch wenn ein negativer Befund eine AD praktisch ausschließt. Eine quantitative Auswertung kann jedoch wertvolle diagnostische Hinweise liefern (Fläche unter der Receiver-operating-characteristics-Kurve [AUC-ROC)] 0,89; C‑Statistik: 0,88) und insbesondere in Kombination mit der [^18^F]FDG-PET (AUC-ROC: 0,94; C‑Statistik: 0,96) bzw. der [^123^I]FP-CIT-SPECT (C-Statistik: 0,98) eine sehr genaue Differenzierung erlauben [33, 63]. Nicht zuletzt besitzt die Amyloid-PET eine sehr hohe Genauigkeit (93 %), um neuropathologisch definierte DLK von DLK/AD und AD zu trennen [64]. Dies kann angesichts des o. g. negativen Effektes von Aβ auf die Kognition (bzw. Erkrankungsprogress) und der ersten FDA-Zulassung von Antiamyloidtherapien perspektivisch von großem therapeutischem Interesse sein.

Aktuelle Tau-PET-Untersuchungen (meist mit [^18^F]AV-1451 bzw. Flortaucipir, als bisher einziger FDA-zugelassener Tracer) zeigen, dass ein substanzieller Anteil der DLK-Patienten eine posterior kortikal betonte Tau-Ablagerung aufweist (z. B. rund 28 % in einer jüngsten, größeren Multicenterstudie 28 % [60]). Insgesamt ist diese aber relativ variabel [65] und geringer ausgeprägt als bei AD [66, 67]. Zudem unterscheidet sich das Verteilungsmuster bei DLK von AD (hier v. a. medial temporal betont [66]) und tritt bei DLK im Gegensatz zur AD auch relativ häufig unabhängig von Amyloidablagerungen auf (A+ T+ 15 %; A− T+ 13 % [60, 65]). Aufgrund o. g. Unterschiede besitzt die Tau-PET möglicherweise eine hohe diagnostische Genauigkeit zur Trennung zwischen DLK und Demenz infolge AD (AUC-ROC: 0,88–0,99; [66, 67]) und scheint insbesondere die klinisch schwierige Differenzierung zwischen DLK und der AD-Variante posteriore kortikale Atrophie (PCA) erheblich zu unterstützen (AUC-ROC > 0,97; [68]).

**Figure 2:**
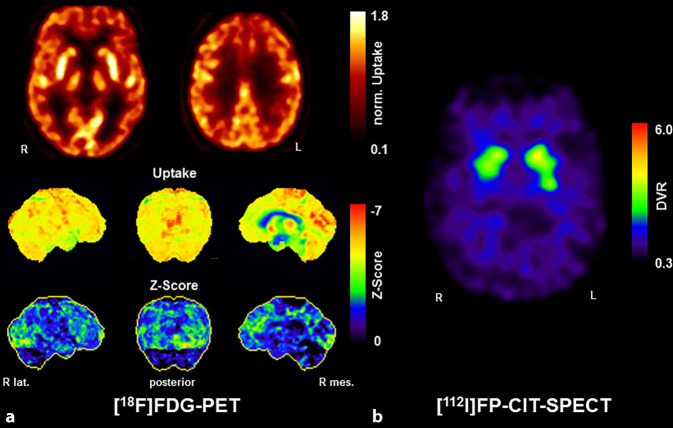

Während der Einsatz von (Bildgebungs‑)Biomarkern zur Diagnostik bei prodromalen Formen (insbesondere „mild cognitive impairment“, MCI) der AD bereits fest etabliert und von großem diagnostischem und prognostischem Nutzen (z. B. [69]) ist, rückt dieser Aspekt bei der DLK mit der kürzlich erfolgten Verabschiedung der Forschungskriterien für prodromale DLK-Formen zunehmend in den Fokus ([8]; Abb. 2). Entsprechende Arbeiten bauen nicht zuletzt auch auf den Ergebnisse von Studien bei RBD-Patienten auf, bei denen typische Befunde z. B. in der [^123^I]FP-CIT-SPECT [10] oder [^18^F]FDG-PET [70] mit einem signifikant erhöhten Konversionsrisiko zu einer klinisch typischen Synucleopathie (PK, MSA oder DLK) einhergingen. Ferner besitzt die [^18^F]FDG-PET auch bei PK-Patienten (mit und ohne MCI) eine gut etablierte prognostische Aussagekraft hinsichtlich einer kognitiven Verschlechterung bzw. Demenz ([57]; z. B. Sensitivität und Spezifität > 85 % für die Prädiktion der Konversion im 4‑Jahres-Follow-up [71]).

Sehr vielversprechende Studienergebnisse liegen nun auch zum neu definierten MCI mit Lewy-Körpern vor (sog. MCI-LK): Sowohl für die [^123^I]FP-CIT-SPECT als auch die [^123^I]MIBG-Szintigraphie wird eine hohe Spezifität (je 88 %) und moderate Sensitivität (66 % bzw. 59 %) für die Trennung zwischen wahrscheinlichem MCI-LK und MCI infolge AD beschrieben [72, 73]. Ähnliche präliminäre Ergebnisse fanden sich für die [^18^F]FDG-PET (AUC-ROC ca. 0,95 [74]). Sehr interessant sind auch erste Ergebnisse einer kombinierten [^123^I]FP-CIT-SPECT- und Amyloid-PET-Studie bei MCI-LK. Diese ergab, dass eine Beteiligung des nigrostriatalen Systems in der DAT-SPECT („D+“) zwar deutlich häufiger als ein Aβ-positiver Befund ist (A− D+, 38 %; A+ D+, 27 %; A− D− 27 %; A+ D− 9 %), aber nur der A+-Status mit schlechterer Kognition korrelierte (ebenso mit höherem Alter und APOE-E4-Status). Die nigrostriatale Integrität korrelierte hingegen mit der Motorik, Fluktuationen und RBD [75]. Inwieweit diese Beobachtungen zur individuellen Risikostratifizierung herangezogen werden können, werden entsprechende Follow-up-Studien zeigen.

## Fazit für die Praxis

Die Diagnose einer DLK wird häufig noch nicht korrekt gestellt; bedingt u. a. dadurch, dass einerseits ein fließender Übergang zwischen der eigentlichen Pathologie mit Lewy-Körpern und einer Alzheimer-Pathologie besteht; andererseits ist bei der Alzheimer-Krankheit das Auftreten der Pathologie mit Lewy-Körpern hoch. Die präzise Beachtung und systematische Anwendung der klinischen Kernmerkmale lassen aber meist eine ätiologische Zuordnung zu; zusätzlich kann die Sensitivität durch den Lewy-Body Composite Risk Score verbessert werden [76]. Es ist zu beachten, dass eine Diagnose auch ohne Parkinson-Symptomatik gestellt werden kann:

- Fluktuierende Bewusstseinslage mit ausgeprägten Schwankungen in Aufmerksamkeit und Wachheit.
- Wiederkehrende visuelle Halluzinationen, die typischerweise Gestaltcharakter haben und detailliert sind.
- REM-Schlafverhaltensstörung, die der kognitiven Störung vorausgehen kann.
- Eine oder mehrere Hauptsymptome der Parkinson-Symptomatik: Bradykinese (definiert als Langsamkeit der Bewegung und Abnahme in der Amplitude oder der Geschwindigkeit), Ruhetremor oder Rigor.
- Eine neuropsychometrische Untersuchung mit besonderer Berücksichtigung der Beeinträchtigungen in der Aufmerksamkeit, exekutiven Funktionen und visuoperzeptiven Fähigkeiten ist erforderlich, während Gedächtnisstörungen nicht unbedingt zu den frühen Symptomen zählen.
- Die Genetik kann derzeit noch keinen wesentlichen Beitrag für die klinische Zuordnung leisten.
- Die bildgebende Zusatzdiagnostik (cMRT, SPECT, PET) unterstützt die Sicherheit der Diagnose einer DLK.
